# Prioritizing direct heart procurement in organ donors after circulatory death does not jeopardize lung transplant outcomes

**DOI:** 10.1016/j.xjtc.2022.08.032

**Published:** 2022-10-06

**Authors:** Stefan Schwarz, Johannes Gökler, Roxana Moayedifar, Clemens Atteneder, Giovanni Bocchialini, Alberto Benazzo, Thomas Schweiger, Peter Jaksch, Andreas O. Zuckermann, Arezu Z. Aliabadi-Zuckermann, Konrad Hoetzenecker

**Affiliations:** a Department of Thoracic Surgery, Medical University of Vienna, Vienna, Austria; b Department of Cardiac Surgery, Medical University of Vienna, Vienna, Austria; c Department of Thoracic Surgery, Department of Medicine and Surgery, University Hospital of Parma, Parma, Italy

**Keywords:** lung transplantation, donation after circulatory death, heart transplantation, CA, circulatory arrest, cDCD, controlled donation after circulatory death, DBD, donation after brain death, ECMO, extracorporeal membrane oxygenation, EVLP, ex vivo lung perfusion, ICU, intensive care unit, ISHLT, International Society for Heart and Lung Transplantation, LTx, lung transplantation, NRP, normothermic regional perfusion, PGD, primary graft dysfunction, PHP, prioritized heart procurement, SWIT, surgical warm ischemic time, WIT, warm ischemic time, WLST, withdrawal of life support therapy

## Abstract

**Background:**

Controlled donation after circulatory death (cDCD) has become a standard in liver, kidney, and lung transplantation (LTx). Based on recent innovations in ex vivo heart preservation, heart transplant centers have started to accept cDCD heart allografts. Because the heart has very limited tolerance to warm ischemia, changes to the cDCD organ procurement procedures are needed. These changes entail delayed ventilation and prolonged warm ischemia for the lungs. Whether this negatively impacts lung allograft function is unclear.

**Methods:**

A retrospective analysis of cDCD lungs transplanted between 2012 and February 2022 at the Medical University of Vienna was performed. The heart + lung group consisted of cases in which the heart was procured by a cardiac team for subsequent normothermic ex vivo perfusion. A control group (lung group) was formed by cases where only the lungs were explanted. In heart + lung group cases, the heart procurement team placed cannulas after circulatory death and a hands-off time, collected donor blood for ex vivo perfusion, and performed rapid organ perfusion with Custodiol solution, after which the heart was explanted. Up to this point, the lung procurement team did not interfere. No concurrent lung ventilation or pulmonary artery perfusion was performed. After the cardiac procurement team left the table, ventilation was initiated, and lung perfusion was performed directly through both stumps of the pulmonary arteries using 2 large-bore Foley catheters. This study analyzed procedural explant times, postoperative outcomes, primary graft dysfunction (PGD), duration of mechanical ventilation, length of intensive care unit (ICU) stay, and early survival after LTx.

**Results:**

A total of 56 cDCD lungs were transplanted during the study period. In 7 cases (12.5%), the heart was also procured (heart + lung group); in 49 cases (87.5%), only the lungs were explanted (lung group). Basic donor parameters were comparable in the 2 groups. The median times from circulatory arrest to lung perfusion (24 minutes vs 13.5 minutes; *P* = .002) and from skin incision to lung perfusion (14 minutes vs 5 minutes; *P* = .005) were significantly longer for the heart + lung procedures. However, this did not affect post-transplantation PGD grade at 0 hours (*P* = .851), 24 hours (*P* = .856), 48 hours (*P* = .929), and 72 hours (*P* = .874). At 72 hours after transplantation, none of the lungs in the heart + lung group but 1 lung (2.2%) in lung group was in PGD 3. The median duration of mechanical ventilation (50 hours vs 41 hours; *P* = .801), length of ICU stay (8 days vs 6 days; *P* = .951), and total length of hospital stay (27 days vs 25 days; *P* = .814) were also comparable in the 2 groups. In-hospital mortality occurred in only 1 patient of the lung group (2.2%).

**Conclusions:**

Although prioritized cDCD heart explantation is associated with delayed ventilation and significantly longer warm ischemic time to the lungs, post-LTx outcomes within the first year are unchanged. Prioritizing heart perfusion and explantation in the setting of cDCD procurement can be considered acceptable.

## Graphical abstract

**Figure:**
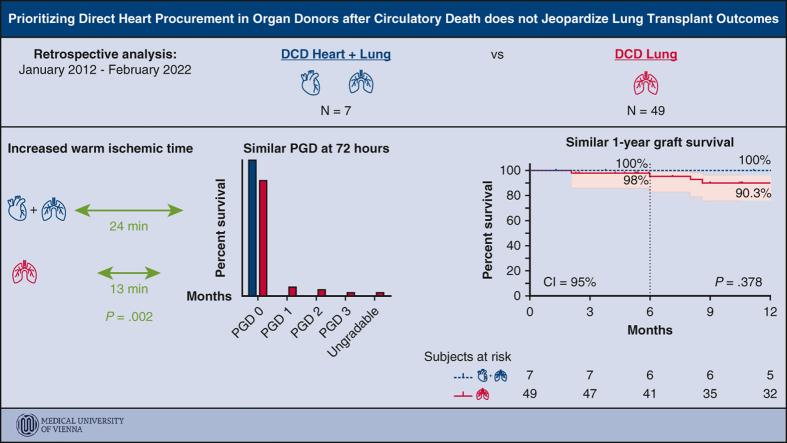

**Figure:**
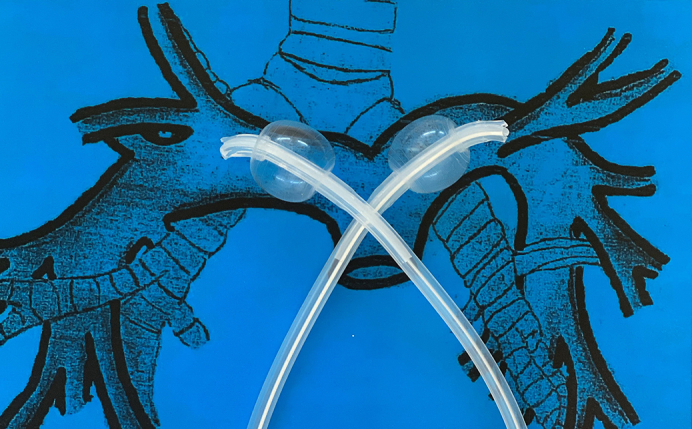
Perfusion of the lung after explantation of the heart.

**Central Message**

Prioritizing direct heart procurement has no detrimental effect on early post–lung transplantation outcomes despite significantly prolonged warm ischemic time and delayed ventilation for the lungs.

**Perspective**

This study provides a first analysis of lung transplantation outcomes using controlled donation after circulatory death (cDCD) donors in which the heart was also explanted using a direct procurement technique. With the increasing use of cDCD hearts, the question arises of whether the heart's warm ischemic time can be safely minimized while accepting prolonged warm ischemia and delayed ventilation for the lungs.

Although organ donation after brain death (DBD) continues to represent the majority of procured organs, controlled donation after circulatory death (cDCD) has successfully expanded the donor pool in recent years and has become a standard in liver transplantation, kidney transplantation, and lung transplantation (LTx). According to the International Society for Heart and Lung Transplantation (ISHLT), cDCD donor lungs are used in 10% of transplantations overall. They account for up to one-third of all LTxs in Australia, Canada, and some European countries but is still less common in the United States, used in only approximately 2% of LTxs.^1^, ^2^, ^3^ Recent innovations have renewed the interest in cDCD heart allografts, and some heart transplantation centers have started to use organs from cDCD donors.^4^, ^5^, ^6^

Currently, 2 basic techniques to retrieve cDCD hearts exist. Direct procurement and ex situ perfusion involves rapid cold perfusion and explantation of the heart, followed by evaluation on a normothermic ex situ machine perfusion device. In contrast, thoracic normothermic regional perfusion (NRP) requires the introduction of extracorporeal circulatory support after declaration of death. Once cardiac action has been reestablished and stabilized, the heart can be procured in a similar manner as in the DBD setting.^4^ Currently, direct procurement is the more common method, owing to logistical issues and ethical as well as legal hurdles for NRP in many countries. The addition of another stakeholder (heart) to the often-hectic setting of cDCD procurement has a potential impact for the other organs (ie, lung, liver, and kidney).

Current consensus recommends simultaneous procedures for all organs in cDCD.^7^ Given the heart's very limited tolerance to warm ischemia, changes to established cDCD organ procurement may be desirable to optimize outcomes. Prioritizing heart perfusion and explantation while delaying lung perfusion provides full access to the thorax for the cardiac team. Delaying ventilation provides an undisturbed surgical field, which is crucial for fast and safe heart dissection in this setting. Recruitment or ventilation of the lungs during this phase causes motion and can obstruct surgical exposure, potentially prolonging warm ischemic time (WIT) for the heart.

The downside of a prioritized heart procurement (PHP) strategy is delayed alveolar aeration and prolonged WIT for the lungs. Currently, there are no data on the consequences of such a practice and whether it negatively impacts lung allograft function; therefore, here we aimed to analyze our early experience with PHP in cDCD lungs.

## Methods

The Institutional Review Board or equivalent Ethics Committee of the Medical University of Vienna approved the study protocol and publication of data (EK-Nr 1951/2020; November 23, 2021). Patient written consent for the publication of the study data was waived because of the study's retrospective nature.

**Figure 1:**
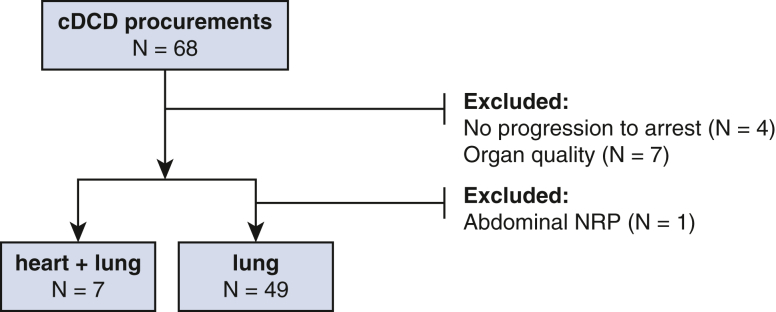
Patient inclusion. *cDCD*, Controlled donation after circulatory death; *NRP*, normothermic regional perfusion.

We retrospectively analyzed cDCD (ie, Maastricht category III) lungs transplanted between January 2012 and May 2021 at the Medical University of Vienna. A total of 56 cDCD lungs were accepted during this time, representing 5.7% of the total center volume. General criteria for organ offer acceptance did not differ between cDCD and DBD donors. Seven donors from whom the heart was also procured were assigned to the heart + lung group. In the remaining 49 donors, the lung was the only thoracic organ procured; these cases composed the lung group and served as controls. One donor with abdominal NRP was excluded from this analysis (Figure 1).

### Definitions

The agonal phase was defined as withdrawal of life support therapy (WLST) to circulatory arrest (CA). The acceptable agonal phase duration for the lungs was limited to 120 minutes. CA was defined as zero systolic arterial blood pressure flatline. WIT was defined as the time between CA and the start of cold lung perfusion. Surgical WIT (SWIT) was defined as the time between skin incision and the start of cold lung perfusion.

### Surgical Procedures

If allowed, heparin (400 U/kg body weight) was given before WLST. In all heart + lung cases, heparin was administered prior to WLST. Bronchoscopy was performed in all cases, either before WLST if allowed by local rules or immediately after declaration of death in parallel to sternotomy and preparation for perfusion.

In lung group cases, the pericardium was opened after rapid sternotomy, and a 22 Fr curved-tip cannula was placed in the main pulmonary artery. Cold perfusion was performed with 6 L of Perfadex (XVIVO Perfusion) supplemented by 1 mg of prostaglandin E1. If the administration of heparin before WLST was prohibited by the local jurisdiction, 10,000 U was added to the perfusion solution.

**Figure E1:**
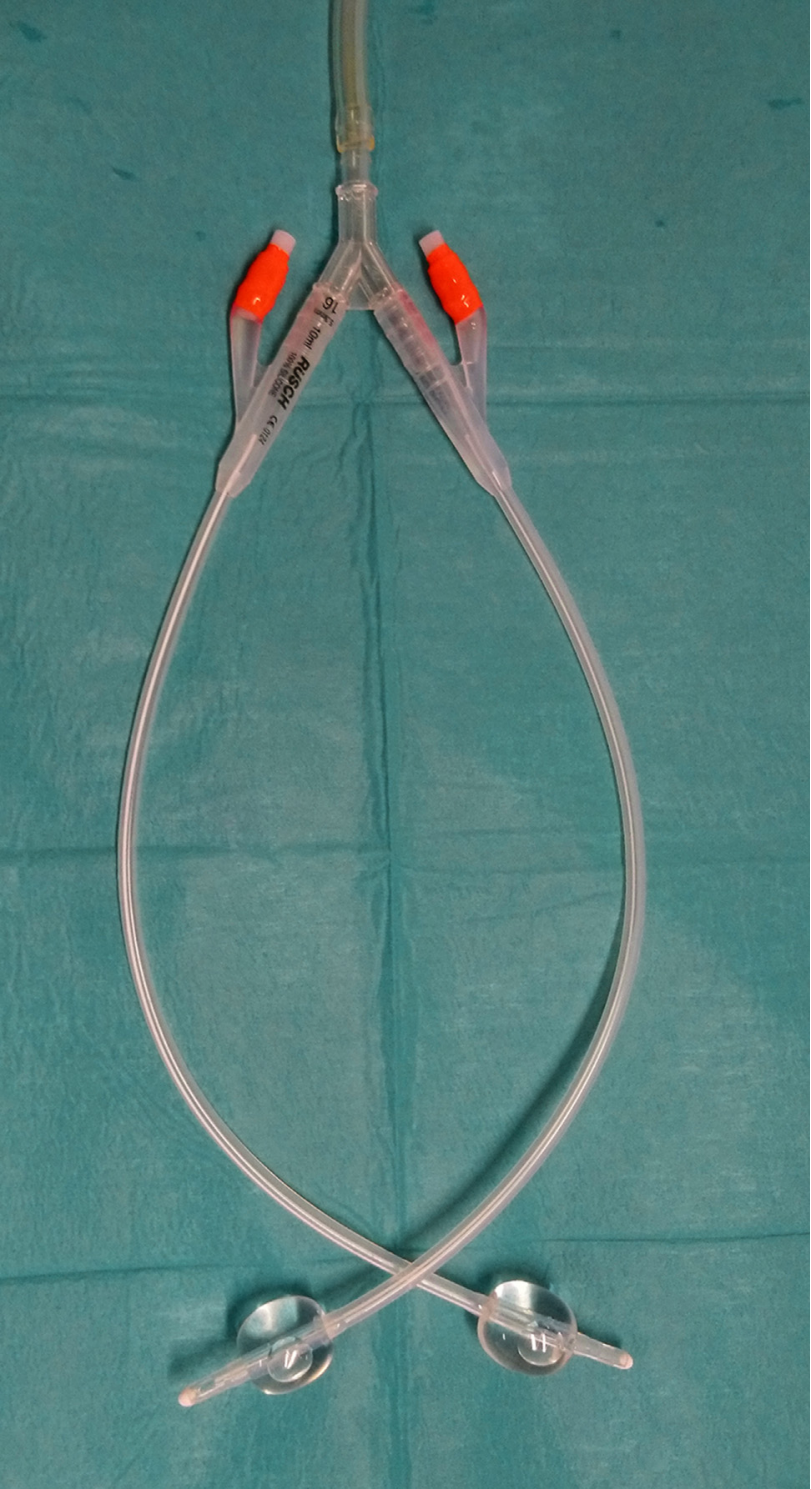
Perfusion set with Foley catheters.

In all heart + lung cases, heart procurement was prioritized. After sternotomy, donor blood was collected for ex vivo perfusion, followed by rapid heart perfusion with 1 L of Custodiol HTK solution (Essential Pharmaceuticals). Explantation of the heart was performed in a standard manner. The lung procurement team was standing by during this first part of the procedure. No concurrent ventilation of the lungs was performed. Once the cardiac procurement team left the table, ventilation was initiated. Lung perfusion using the solution and additives described above was performed by inserting 2 cuffed large-bore (18-20 Fr) Foley catheters directly into the left and right pulmonary arteries via the opened arterial bifurcation (Figure E1).

Following cold lung perfusion, standard procurement was performed as described elsewhere.^8^ After explantation, retrograde flushing was performed with 1 L of Perfadex through the left atrial cuff. For the implantation, we followed our institutional standard procedure, using central veno-arterial extracorporeal membrane oxygenation (ECMO) in all cases.^9^ If defined criteria for early organ function were not met at the end of the procedure, the ECMO was switched to a femoro-femoral veno-arterial configuration and prophylactically prolonged into the postoperative period.

### Recipient Data

Recipient data as well as perioperative and follow-up data were retrieved from our institutional databases. Radiological assessment for primary graft dysfunction (PGD) grading was provided by trained chest radiologists.

### Outcome Parameters

Early recipient outcome analysis included results within the first year post-transplantation. PGD grades at 24, 48, and 72 hours were assessed according to current ISHLT guidelines.^10^ Patients with postoperatively prolonged ECMO support were graded as PGD 3 or PGD “ungradable” depending on the chest X-ray results. Total duration of mechanical ventilation was defined as the time to successful extubation without early reintubation (<3 days). In cases with tracheostomy, duration of mechanical ventilation was defined as the time when the patient tolerated mere oxygen insufflation without any mechanical breathing assistance for >6 continuous hours. Furthermore, lengths of intensive care unit (ICU) and total hospital stay, postoperative complications, in-hospital mortality, airway complications, and 1-year survival were determined. Histologically verified acute rejections of both perivascular (A-grading) and airway (B-grading) forms were evaluated and reported in cases of grade 2 or greater. The highest value of forced expiratory volume at 1 second achieved during follow-up was calculated as the percentage of the predicted value for each respective recipient.

### DBD Cohort

Donor and recipient demographic data were further compared between cDCD transplants (n = 56) (combining the heart + lung and lung groups) and a matched, contemporary cohort of DBD transplants (n = 165) as controls.

### Statistical Analysis

Statistical analysis was performed with SPSS 26 (IBM). A *P* value <.05 was considered statistically significant. Missing data were coded appropriately, and cases were excluded from the respective analysis. Continuous variables were compared using the *t*-test or Mann–Whitney U test according to data distribution. The χ^2^ test or Fisher exact test were used for categorical variables as applicable. Figures were created with Prism 8 (GraphPad Software). Propensity score matching was performed using R software (R Foundation for Statistical Computing) using the “MatchIt” package. Matching of all cDCD cases (the heart + lung and lung groups combined) to a contemporary control group of DBD cases (1:3) was performed using recipient age, body mass index, underlying diagnosis, and wait list urgency as covariates. Greedy matching with a caliper set at 0.3 was performed.

## Results

### Donor Demographics

Basic donor characteristics are detailed in Table 1. The majority of donors in the heart + lung group suffered from isolated head trauma (58%); 1 patient (14%) had a cerebrovascular incident, and 1 patient (14%) had prolonged status asthmaticus. In 1 case (14%) in which the donor had sustained CA before regaining spontaneous circulation, the donor heart was eventually rejected after evaluation on EVP. Heart + lung donors had received cardiopulmonary resuscitation more often (n = 6; 86%) compared with lung donors (n = 20; 42%). Median Oto scores were similar at 4 (IQR, 2-6) in the heart + lung group and 5 (IQR, 3-6) in the lung group. Most donor parameters were comparable between the heart + lung group and the matched DBD control group. Cerebrovascular incidents were significantly more prevalent in the control group (69%), whereas isolated head trauma was more common in the heart + lung group (18%) (*P* < .001). The mean last donor PaO_2_ value was higher in the control group (428 ± 104 mm Hg vs 391 ± 94 mm Hg; *P* = .017).

**Table 1:** Donor characteristics

Characteristic	Heart + lung group (N = 7)	Lung group (N = 49)	*P* value	Matched DBD controls (N = 165)	*P* value
Age, y, median (IQR)	42 (28-48)	53 (42-58)	.342	48 (36-56)	.770
Sex, male/female, %	42.9/57.1	59.2/40.8	.447	41.2/58.8	.044∗
Height, cm, median (IQR)	175 (165-180)	175 (168-180)	.771	170 (165-180)	.982
Weight, kg, median (IQR)	76 (70-90)	74.5 (70-85)	.436	74 (65-83)	.185
BMI, median (IQR)	26 (21-29)	25 (23-27)	.562	25 (22-27)	.969
Blood group, n (%)					
A	1 (14.5)	22 (45)	.237	61 (37)	.535
B	1 (14.5)	2 (4)		19 (11)	
O	5 (71)	21 (43)		77 (47)	
AB	0 (0)	4 (8)		8 (5)	
Cause of condition, n (%)					
Cardiac incident	1 (14)	8 (16)	.089	7 (4)	<.001∗
Cerebrovascular incident	1 (14)	23 (48)		114 (69)	
Isolated head trauma	4 (58)	6 (12)		13 (8)	
Polytrauma	0 (0)	3 (6)		10 (6)	
Other	1 (14)	9 (18)		21 (13)	
CPR, n (%)	6 (86)	20 (42)	.044∗	43 (26)	.005∗
Oto score >7, n (%)	0 (0)	4 (9)	.999	22 (14)	.217
Intubation, d, median (IQR)	7 (3-8)	4 (2-8)	.463	4 (2-6)	.232
Last PaO_2_, mm Hg, mean ± SD	409 ± 82	387 ± 97	.687	428 ± 104	.017∗
Last PaCO_2_, mm Hg, mean ± SD	38 ± 7	40 ± 8	.705	39 ± 8	.486

*DBD*, Donation after brain death; *IQR*, interquartile range; *BMI*, body mass index; *CPR*, cardiopulmonary resuscitation.

∗ Significant *P* value (*P* < .05).

### Organ Procurement

**Figure 2:**
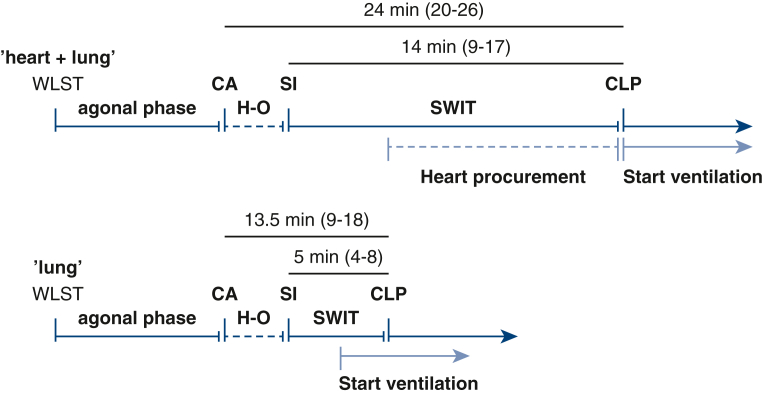
Procurement procedure. *WLST*, Withdrawal of life-sustaining therapy; *CA*, circulatory arrest; *SI*, skin incision; *CLP*, cold lung perfusion; *H-O*, hands-off time; *SWIT*, surgical warm ischemic time.

The time sequences of organ procurement for the heart + lung and lung groups are depicted in Figure 2. The median duration of the agonal phase was similar in the 2 groups (12 minutes vs 11 minutes; *P* = .937). The time between CA and cold lung perfusion (ie, WIT) included different hands-off periods, ranging from 3 to 10 minutes. The median WIT was 24 minutes (IQR, 20-26 minutes) in the heart + lung group, compared with 13.5 minutes (IQR, 9-18 minutes) in the lung group (*P* = .002). To correct for varying hands-off times across donor site jurisdictions, we also calculated SWIT as defined above. The median SWIT was 14 minutes (IQR, 9-17 minutes) in the heart + lung group, compared with 5 minutes (IQR, 4-8 minutes) in the lung group (*P* = .005).

### Recipient Demographics

Recipient data are summarized in Table 2. The 2 patient groups were similar in age (*P* = .511), sex distribution (*P* = .700), and primary diagnosis (*P* = .552). Median lung allocation scores were similar in the heart + lung and lung groups at 32.6 (IQR, 32-34) and 37.4 (IQR, 33-63), respectively. A comparable proportion of patients in the 2 groups were ventilator-dependent immediately before transplantation (*P* = .999) or required pretransplantation extracorporeal bridging therapy (*P* = .999). One patient (lung group) underwent transplantation on cardiopulmonary bypass to correct a giant pulmonary artery aneurysm as described elsewhere^11^; all other patients received routine intraoperative central veno-arterial ECMO as described above. Patients in the heart + lung group required a median of 2.5 units (IQR 2-4.5 units) of packed red blood cells and a median of 5.5 units (IQR 4-8 units) of fresh frozen plasma. In the lung group, a median of 5 units (IQR, 2-8 units) of packed red blood cells and 10 units (IQR, 8-13 units) of plasma concentrate were used (*P* = .012 and .049, respectively). This difference may be explained by the higher proportion of patients with cystic fibrosis and primary pulmonary hypertension in the lung group. Six lung group patients (12%) but no heart + lung group patients required postoperative veno-arterial ECMO due to impaired early organ function according to our above-described protocol (*P* = .999).

**Table 2:** Recipient characteristics

Characteristic	Heart + lung group (N = 7)	Lung group (N = 49)	*P* value	Matched DBD controls (N = 165)	*P* value
Age, y, median (IQR)	56 (38-61)	55 (36-61)	.511	53 (36-61)	.817
Sex, male/female, %	43/57	53/47	.700	51/49	.910
Diagnosis, n (%)					
COPD	5 (72)	20 (42)	.552	73 (44)	.983
Fibrosis	1 (14)	7 (15)		28 (17)	
Cystic fibrosis	0 (0)	11 (24)		35 (21)	
Primary pulmonary hypertension	0 (0)	3 (6)		6 (4)	
A1AD	0 (0)	1 (2)		3 (2)	
Other	1 (14)	5 (11)		20 (12)	
Lung allocation score, median (IQR)	32.6 (32-34)	37.4 (33-63)	.801	36.4 (33-49)	.962
Transplant type (n, %)					
Full	5 (71)	24 (49)	.525	94 (57)	.661
Size reduced	2 (29)	24 (49)		64 (39)	
Lobar	0 (0)	1 (2)		7 (4)	
Ischemic time, min, mean ± SD	458 ± 239	414 ± 98	.748	365 ± 65	.002∗
ECLS bridging, n (%)	1 (14)	9 (18)	.999	17 (10)	.136
Intubated pretransplant, n (%)	1 (14)	7 (15)	.999	11 (7)	.098
Type of intraoperative support, n (%)					
No support	0 (0)	0 (0)	.883	3 (2)	.629
Intraoperative ECMO	7 (100)	48 (98)		158 (95)	
CPB	0 (0)	1 (2)		4 (3)	
Intraoperative pRBC units, median (IQR)	2.5 (2-4.5)	5 (2-8)	.012∗	4 (3-8)	.944
Intraoperative FFP units, median (IQR)	5.5 (4-8)	10 (8-13)	.049∗	10 (7-14)	.963
Prolonged postoperative VA ECMO, n (%)	0 (0)	6 (12)	.999	21 (13)	.691
Duration of MV, h, median (IQR)	50 (30-68)	41 (25-76)	.801	42 (21-96)	.870
ICU stay, d, median (IQR)	8 (4-11)	6 (4-20)	.951	8 (5-15)	.058
Hospital stay, d, median (IQR)	27 (25-37)	25 (20-41)	.814	30 (21-47)	.415

*DBD*, Donation after brain death; *IQR*, interquartile range; *COPD*, chronic obstructive pulmonary disease; *A1AD*, alpha-1 antitrypsin deficiency; *SD*, standard deviation; *ECLS*, extracorporeal circulatory support; *ECMO*, extracorporeal membrane oxygenation; *CPB*, cardiopulmonary bypass; *pRBC*, packed red blood cell; *FFP*, fresh frozen plasma; *VA*, veno-arterial; *MV*, mechanical ventilation; *ICU*, intensive care unit.

∗ Significant *P* value (*P* < .05).

Most recipient demographic parameters were similar in the combined DBD group and the matched DBD control group. The mean graft ischemic time was significantly shorter for DBD controls, at 365 ± 65 minutes (*P* = .002). This most likely can be explained by the travel distance between Vienna and centers in Belgium and The Netherlands with the highest frequency of cDCD donation.

### Outcomes

**Figure 3:**
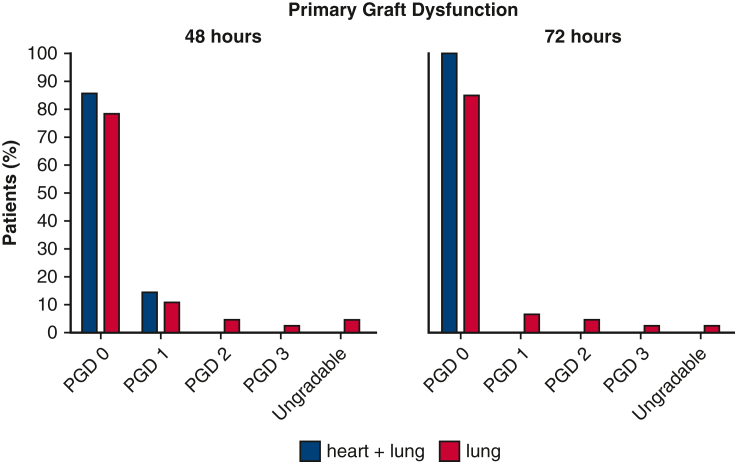
Primary graft dysfunction score. *PGD*, Primary graft dysfunction.

**Figure E2:**
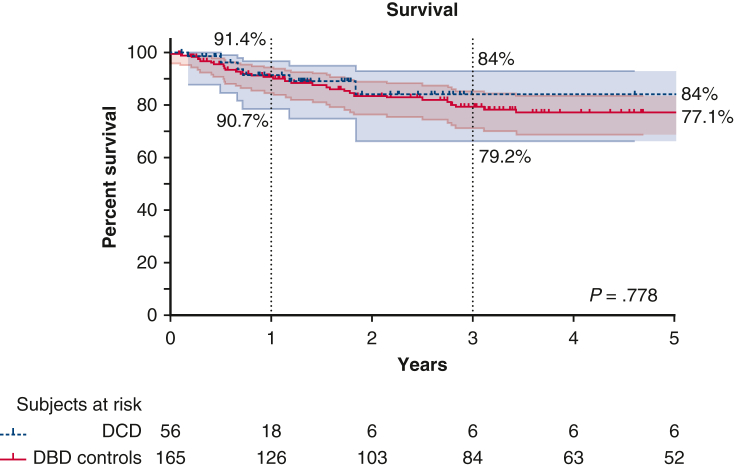
Five-year survival compared to matched controls. *Shaded areas* represent 95% CIs. *DCD*, Donation after circulatory death; *DBD*, donation after brain death.

Rates of PGD were similar at all time points in both groups (Figure 3). At 72 hours, all 7 patients in the heart + lung group were classified as PGD 0. In the lung group, the majority were graded as PGD 0 (n = 39; 84.8%), and 3 (6.5%) were graded as PGD 1, 2 (4.3%) as PGD 2, and 1 (2.2%) as PGD 3. One patient (2.2%) was ungradable, with a clear chest X-ray while still on veno-arterial ECMO (*P* = .874). Patients required mechanical ventilation for a median of 50 hours (IQR, 30-68 hours) in the heart + lung group versus 41 hours (IQR, 25-76 hours) in the lung group (*P* = .801). The length of postoperative stay in the ICU was similar in the 2 groups, with a median of 8 days (IQR, 4-11 days) in the heart + lung group versus 6 days (IQR, 4-20 days) in the lung group (*P* = .951). The median length of hospital stay also was comparable in the 2 groups (27 days vs 25 days; *P* = .814). The median duration of mechanical ventilation and lengths of postoperative ICU stay and total hospital stay were similar in the combined cCDC group and the DBD control group (*P* = .870, .580, and .415, respectively). No significant difference in 5-year survival was found between the cDCD and DBD groups (Figure E2).

**Figure 4:**
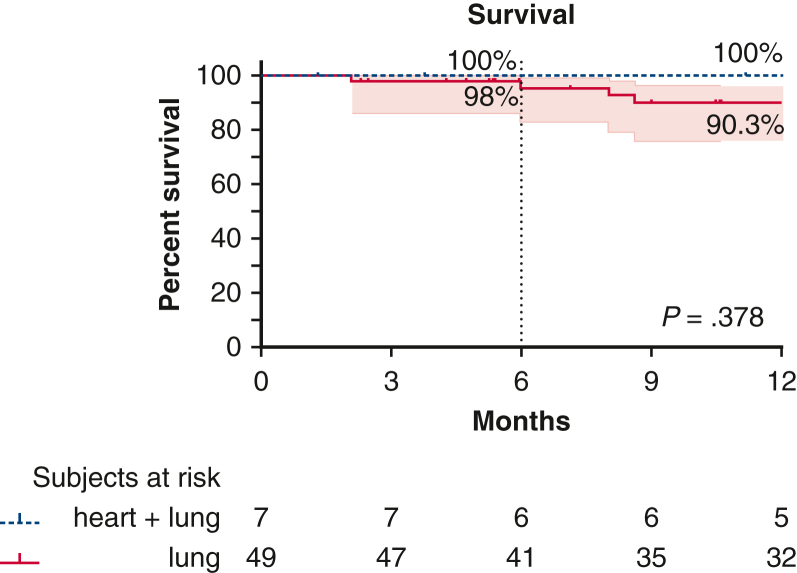
One-year survival. *Shaded areas* represent 95% CIs.

**Figure 5:**
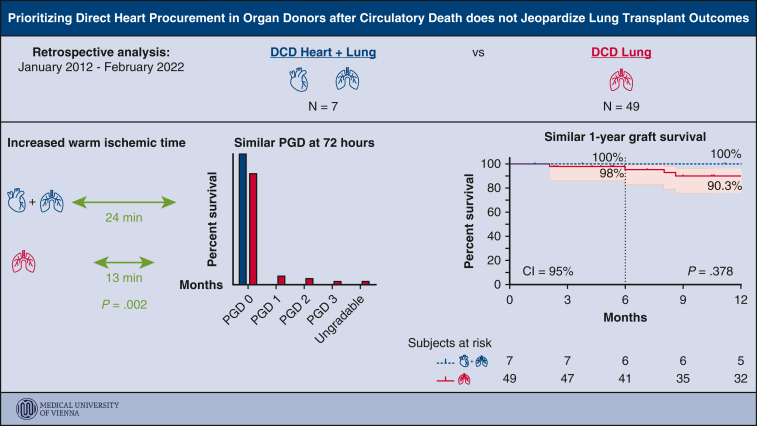
Overview of study rationale and outcomes. *DCD*, Donation after circulatory death; *PGD*, primary graft dysfunction; *CI*, confidence interval.

Only 1 patient (in the lung group) died in the early postoperative course, resulting in an in-hospital mortality rate of 2.0%, compared with to 0% in the heart + lung group (*P* = .999). One-year survival was comparable in the 2 groups, at 100% for the heart + lung group and 90.3% for the lung group (*P* = .378) (Figure 4). One patient (2%) in the lung group developed an airway complication; this case of bronchial dehiscence of the right main bronchus was found at 3 months post-transplantation and resolved without the need for intervention. Rates of acute perivascular rejection ≥ A2 (heart + lung, 0%; lung, 4.1%; *P* > .999) and acute airway rejection ≥ B2 (heart + lung, 14.3%; lung, 0%; *P* = .125) did not differ between the 2 groups. The best forced expiratory volume at 1 second values reached during follow-up were 85.6% of the predicted value in the heart + lung group and 82.9% in the lung group (*P* = .911). This peak value was reached after a median of 114 days in the heart + lung group and 166.5 days in the lung group (*P* = .443). The study rationale and important outcomes are summarized in Figure 5 and Video 1.

## Discussion

cDCD heart transplantation is an emerging practice with excellent results and has the potential to significantly increase heart transplant activity.^12^ However, data on the functional outcome of the lungs from cDCD heart donors is lacking. To the best of our knowledge, this study represents the first analysis of outcome in this group of cDCD donors. Moreover, it is the first to examine the practice of prioritizing heart procurement while delaying both lung perfusion and ventilation. These results of our early experience suggest that (1) the delay of lung inflation and cold perfusion by PHP is typically limited to 10 to 15 minutes, and (2) the use of this protocol has no effect on early lung performance.

Messer and colleagues^6^ were the first to mention the potential advantages of the PHP strategy in cDCD heart procurement. In the United Kingdom, the most recent national protocol for cDCD procurement of heart and lungs now recommends PHP with early lung reinflation and delayed cold perfusion. This decision was made despite concerns raised by some LTx representatives.^13^^,^^14^ The major advantages of a PHP strategy for the heart are obvious: reduced WIT, better surgical exposure, and reduced cold ischemic time without the need to wait for completion of pulmonary perfusion. Donor management and multiorgan procurement have always required balancing contrary interests to ensure success for many stakeholders. In cDCD, where aspects contributing to WIT are not modifiable, steps of the surgical procurement procedure become even more important. This is illustrated by ongoing discussions about potential delays of 90 to 120 seconds in initiating cold perfusion in combined cDCD liver and heart explantation.^15^^,^^16^ When sharing the even smaller operating field of the chest, procedural aspects clearly are even more important. Establishing evidence-based guidelines is essential to safely expand the use of cDCD hearts.

A large body of evidence indicates that outcomes of cDCD LTx are equal to those of DBD LTx.^2^^,^^17^, ^18^, ^19^, ^20^, ^21^ Importantly, none of the available reports on cDCD lungs state whether other organs were procured. Given the only-recent initiation of cDCD heart transplant programs, it can be assumed that these reports did not include any donors in which the heart was used. Although data on combined heart and lung cDCD procurement is lacking, several previous studies have aimed to identify limits for acceptable cDCD lungs. These studies also can be used to evaluate the impact of prioritizing heart explantation on cDCD lung donation.

The agonal phase is usually defined as the time from WLST to CA. In our study, this interval was significantly shorter in the heart + lung group than in the lung group. The difference is most likely explained by the restrictive time limit of 15 minutes of agonal phase for cDCD heart procurement to prevent potentially irreversible damage to the heart. The Vienna Heart Transplant Program currently accepts a maximum agonal phase of 15 minutes before abstaining from heart procurement. For the lungs, an agonal time of up to 120 minutes is considered acceptable by our center. Based on a registry report published by Cypel and colleagues,^1^ internationally this center-specific time frame ranges from 30 to 180 minutes. Single-center studies as well as a multicenter study have shown no impact of agonal phase duration on LTx outcomes within these limits.^19^^,^^22^ This wide range that LTx centers are willing to accept while reporting excellent outcomes demonstrates confidence in the lungs' tolerance to warm ischemia.

As our results show, WIT of the lungs is significantly prolonged by the addition of PHP. An ISHLT working group has recommended standardized time points and intervals for cDCD reporting^1^; however, definitions in the literature for the start of WIT still vary among WLST, hemodynamic instability, and CA.^23^ Currently, ISHLT interval 4 (systolic blood pressure <50 mm Hg to cold perfusion), sometimes termed functional warm ischemia, is usually considered the most relevant definition.

The extent to which WIT impacts cDCD lung outcomes remains controversial. Impaired early oxygenation capacity of transplanted cDCD lungs with a prolonged time between low blood pressure (<50 mm Hg) and CA has been reported.^24^ On the other hand, Levvey and colleagues^22^ found no impact of functional warm ischemia, defined as donor systolic blood pressure <50 mm Hg to initiation of cold perfusion, on early survival in a multicenter registry study. A greater risk of airway complications for cDCD lungs has been postulated owing to hypoperfusion of the main bronchi during functional WIT.^25^^,^^26^ Thus, based on this consideration, extending WIT by PHP could further increase the risk for anastomotic problems. In our cohort, only 1 bronchial complication was recorded in the lung group, and none were seen in the heart + lung group; however, this also could be related to the generally low complication rate with a single running suture technique.^27^

An important contributor to warm ischemia is the so-called “hands-off” time. This period is highly variable between jurisdictions, ranging from 2 minutes in Australia to 10 minutes in most parts of Europe and even 20 minutes in Italy.^1^^,^^4^ Hands-off times in our present cohort ranged from 3 to 10 minutes. Because this interval is unmodifiable, we instead focused on the time from skin incision to cold perfusion in our analyses. Although the skin incision to perfusion time was comparable with published data in the lung group, it was significantly longer in the heart + lung group. Heart procurements in our series were performed by different cardiac teams from multiple institutions; however, all were highly experienced in the explant procedure. Topical cooling is a possible strategy to potentially reduce some of the effects of warm lung ischemia while the heart is being procured. We aimed to provide the cardiac teams with optimal procurement conditions and thus abstained from any measures that could impair surgical exposure and delay the procedure in any way.

The lungs are known to have the unique ability to maintain oxygen supply to the parenchyma through passive diffusion from the alveoli even in absence of perfusion. The protective effect of inflation during normothermic lung ischemia on adenosine triphosphate stores and lactate generation has been described by De Leyn and associates.^28^ This factor has been used by Italian centers in light of their mandatory 20- minute hands-off period. They instead have focused on in situ preservation by alveolar recruitment while facilitating abdominal NRP. According to a case series reported by Palleschi and colleagues,^29^ the Milan group incurred WITs of 80 to 250 minutes from asystole to pulmonary flush. Four lungs were procured and were subjected to ex vivo lung perfusion (EVLP); 3 of these lungs were found to be suitable, whereas the organ with the longest WIT was rejected after EVLP. Of note, no topical cooling was performed with this technique, in contrast to the in situ preservation protocols that other groups have proposed.^30^ Animal models have shown that the prevention of alveolar collapse, rather than ventilation or fraction of inspired oxygen, may protect the lungs during warm ischemia.^31^^,^^32^ In a series of uncontrolled DCD LTxs, the Toronto group used mere inflation with a continuous positive airway pressure of 20 cmH_2_O to protect the organ during a WIT of 106 to 199 minutes, yielding excellent results.^33^ They explained the utilization rate of only 15% by the negative effects of warm ischemia without concurrent lung inflation in the time necessary to obtain family consent. To provide the cardiac team with optimal undisturbed surgical exposure, we accepted delayed initiation of lung inflation and ventilation during warm ischemia for a PHP of 10 to 15 minutes until the heart was explanted. We did not find any detrimental impact on post-transplantation outcomes with this approach. Although moderate continuous pressure or ventilation with regular tidal volumes could impair surgical conditions for heart procurement, early alveolar recruitment, continuous low or very low tidal volume ventilation may be a feasible lung protective strategy to overcome concerns regarding PHP. In the event of doubts about the organ quality of cDCD lungs, EVLP is an excellent method for assessing the lungs and potentially repairing a primarily unacceptable organ in the future. In our current cDCD protocol, EVLP is not mandatory but is used only in selected cases to evaluate grafts of questionable quality. In this series, none of the lungs in the heart + lung group but 2 lungs (4.0%) in the lung group required EVLP evaluation.

Our study has several limitations. It is a retrospective analysis, which comes with the possibility of miscoded data and missing parameters. Consensus on the systolic blood pressure threshold to define insufficient organ perfusion and represent the start of functional warm ischemia remains lacking.^34^^,^^35^ Inconsistencies in the reporting of this parameter (40, 50, or 60 mm Hg) prevented us from meaningfully calculating comparable functional WITs for our cohort. Because our study covers a practice that only recently transitioned from experimental to routine, the cohort size for PHP is limited, and it is too early to report on long-term outcomes. The sample size also limits the possibility of statistically adjusting for differences between the groups. In addition, the risk for a type II error may be increased. As a single-center analysis, our study provides a homogenous cohort of a high-volume program and offers good data granularity; nonetheless, multicenter or registry studies examining this topic would be beneficial. Furthermore, because our study covers an extended time frame, and increased heart cDCD is a recent development, era effects cannot be ruled out.

In conclusion, this study of our early experience shows that PHP in cDCD is associated with delayed ventilation and prolonged WIT for the lungs. However, prioritizing heart perfusion and explantation in the setting of cDCD procurement did not affect early post-LTx outcomes.

### Webcast

You can watch a Webcast of this AATS meeting presentation by going to: https://www.aats.org/resources/1733.

**Figure:**
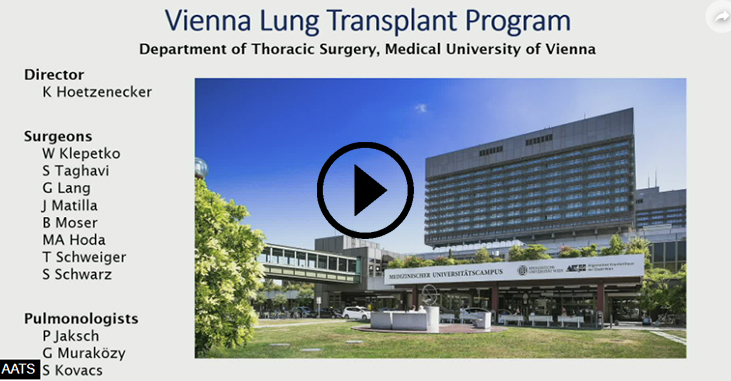

### Conflict of Interest Statement

The authors reported no conflicts of interest.

The *Journal* policy requires editors and reviewers to disclose conflicts of interest and to decline handling or reviewing manuscripts for which they may have a conflict of interest. The editors and reviewers of this article have no conflicts of interest.

## Supplementary Data

Video 1 Description of the study rationale and basic results. Video available at: https://www.jtcvs.org/article/S2666-2507(22)00523-5/fulltext.
