# Supplementary figures and images for: Prioritizing direct heart procurement in organ donors after circulatory death does not jeopardize lung transplant outcomes
Source: JTCVS Tech. 2022 Oct 6;16:182–95. doi: 10.1016/j.xjtc.2022.08.032 (PMC9737044; doi:10.1016/j.xjtc.2022.08.032)

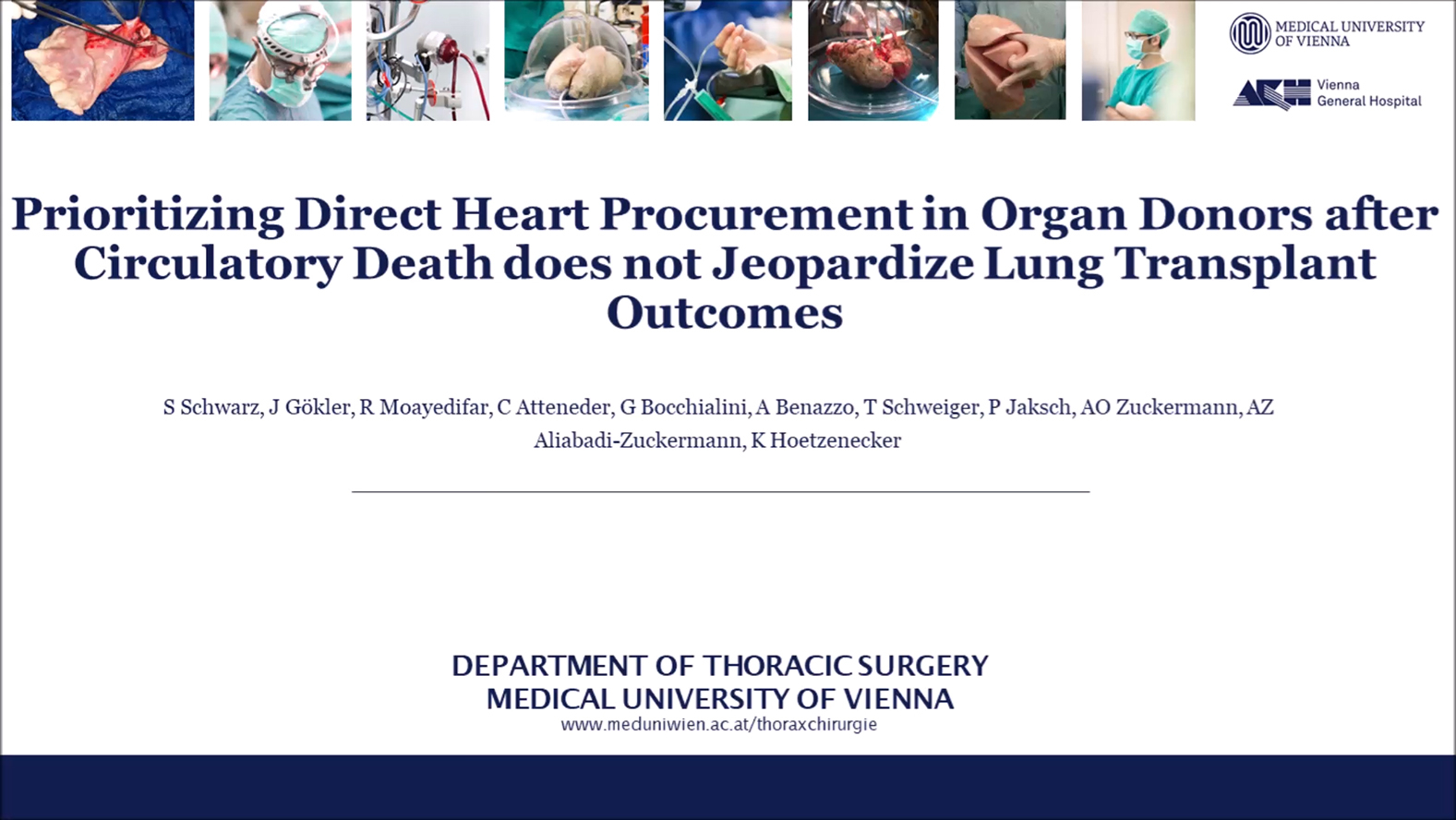

Supplement: Video 1 — Description of the study rationale and basic results. Video available at: https://www.jtcvs.org/article/S2666-2507(22)00523-5/fulltext. [file fx4.jpg]
